# STOPDAPT-3 subanalysis on prasugrel monotherapy after elective or emergent coronary intervention in patients with or without diabetes: are we ready for this?

**DOI:** 10.1093/ehjcvp/pvae079

**Published:** 2024-11-19

**Authors:** Jeehoon Kang, Giuseppe Gargiulo

**Affiliations:** Cardiovascular Center, Seoul National University Hospital, 101 Deahakro, Chongno-gu, Seoul 03080, Republic of Korea; Department of Advanced Biomedical Sciences, Federico II University of Naples, Via Pansini 5, 80131, Naples, Italy

**This editorial refers to, ‘An aspirin-free strategy for percutaneous coronary intervention in patients with diabetes: a pre-specified subgroup analysis of the STOPDAPT-3 trial’, by K. Yamamoto *et al.*  https://doi.org/10.1093/ehjcvp/pvae075**.

Dual antiplatelet therapy (DAPT) is the foundation of pharmacologic treatment to prevent thrombotic or ischaemic events during and after percutaneous coronary intervention (PCI), but its benefits come at the expense of an increased risk of bleeding complications.^1^ Reducing (i.e. de-escalation) the intensity of antiplatelet therapy after PCI, including the aspirin-free strategies, is a relevant alternative emerged and has been investigated in the last decade to mitigate the bleeding risk, particularly in patients at high bleeding risk (HBR).^2^ This strategy is possible due to the improvements in PCI procedures, coronary stents technologies and the optimal medical treatments that help reducing the ischaemic risk. However, softening antiplatelet therapy cannot be applied to all patients, while the ischaemic and bleeding risks should be meticulously evaluated. Meanwhile, it is well-known that patients with diabetes mellitus (DM) are characterized by enhanced thrombotic risk attributed to multiple mechanisms including hyper-reactive platelets, hypercoagulable status, and endothelial dysfunction (*Figure 1*). Thus, they are more prone to atherosclerotic cardiovascular events than patients without DM, both before and after coronary artery disease is established.^3,4^ In patients with DM who receive PCI, the metabolic alterations that occur by hyperglycaemia can accelerate the formation of neointimal hyperplasia. Also, DM induces drug resistance and hypersensitivity to the stent, which increases the risk of delayed endothelialization and restenosis,^5^ as well as acute events such as stent thrombosis.^6^ Based on the cardiovascular risk according to DM, antiplatelet agents are a cornerstone of preventing adverse clinical events in patients with diabetes. Consequently, we can find that the recommended DAPT strategy is slightly more intense in DM patients, compared to the general population.^4,7^ However, in a large population of more than 11 000 patients from 6 randomized trials of different DAPT regimens, despite DM was an independent predictor of adverse cardiovascular events after coronary stenting with a drug-eluting stent, long-term compared with short-term DAPT did not reduce the risk of such events while increased the risk of bleeding among patients with and without diabetes.^8^ Yet, despite it is well-known that DM is characterized by a prothrombotic milieu affecting the response to clopidogrel, a higher platelet reactivity has been also described for the more potent ticagrelor and prasugrel in patients with DM compared with those without.^9^ However, in the TWILIGHT trial, ticagrelor monotherapy reduced the risk of bleeding without a significant increase in ischaemic events compared with DAPT (ticagrelor plus aspirin) irrespective of the presence of DM, chronic kidney disease, and their combination.^10^

**Figure 1:**
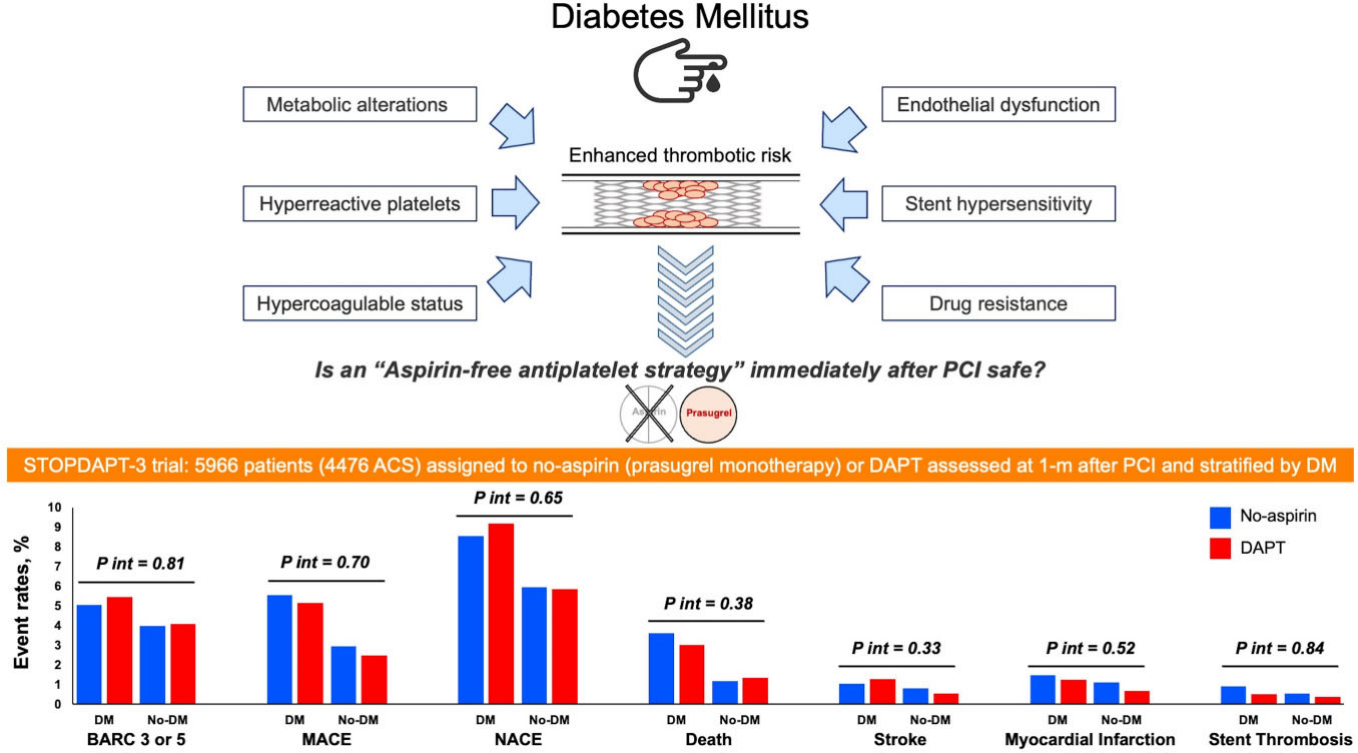
Diabetes mellitus and clinical outcomes of the STOPDAPT-3 trial. ACS, acute coronary syndrome; BARC, Bleeding Academic Research Consortium; DAPT, dual antiplatelet therapy; DM, diabetes mellitus; MACE, major adverse cardiovascular events; NACE, net adverse clinical events; PCI, percutaneous coronary intervention. Notes: MACE definition in the trial (co-primary cardiovascular endpoint): composite of cardiovascular death, myocardial infarction, definite stent thrombosis, or ischaemic stroke. NACE definition (major secondary endpoint): composite of cardiovascular death, myocardial infarction, definite stent thrombosis, ischaemic stroke, or BARC 3 or 5 bleeding.

In the current issue, Yamamoto *et al.* evaluated the impact of ultrashort DAPT duration after PCI in patients with and without DM.^11^ This study was a pre-specified subgroup analysis of the STOPDAPT-3 trial, which compared 1-month prasugrel monotherapy without aspirin with 1-month DAPT consisting of aspirin and prasugrel.^12^ The co-primary bleeding endpoint was Bleeding Academic Research Consortium type 3 or 5 bleeding events, and the co-primary cardiovascular endpoint was a composite of cardiovascular death, myocardial infarction, definite stent thrombosis, or ischaemic stroke at 1-month post-PCI (*Figure 1*). Among the total population of 5966 patients, 2715 patients (45.5%), and 3251 patients (54.5%) were with or without DM, respectively. Overall, DM patients had a higher risk for both co-primary endpoints. When comparing the two antiplatelet strategies, no significant interaction was observed for both the co-primary bleeding endpoint (*P* for interaction = 0.81) or the co-primary cardiovascular endpoint (*P* for interaction = 0.70). Even in patients who presented with acute coronary syndrome (approximately 75% of the study population), no significant interaction was observed between the treatment strategy and DM. The study findings imply that the impact of the aspirin-free prasugrel monotherapy strategy was consistent regardless of the diabetic status, in terms of cardiovascular and bleeding endpoints.

The STOPDAPT-3 trial is innovative, and the authors should be commended for providing this additional analysis dedicated to a subgroup of peculiar interest. However, although the numbers seem to support the safety of aspirin-free strategy in DM patients, a few important points should be considered. First, the original design of STOPDAPT-3 trial should be taken into account when interpreting the results of the present substudy. The original hypothesis of the study was to prove superiority of the bleeding endpoint and non-inferiority of the cardiovascular endpoint for the prasugrel monotherapy arm as compared to the DAPT arm. The results at 1-month showed that the prasugrel monotherapy arm was not superior to the DAPT arm for the co-primary bleeding endpoint (4.47% vs. 4.71%; HR, 0.95; *P* for superiority = 0.66), while the 1-month co-primary cardiovascular endpoint met non-inferiority (4.12% vs. 3.69%; HR, 1.12; *P* for non-inferiority = 0.01). Unlike the expectation that removal of aspirin would significantly reduce the bleeding risk, this was associated with only 5% relative risk reduction in major bleeding. This can be explained by the HBR of the study population, and the impact of other antithrombotic agents in the periprocedural period, which outweigh the impact of aspirin. Irrespective of the reason for a negative result, caution is needed when a subgroup analysis is performed from a negative study. It is not only unlikely to derive significant findings, but also the potential type 1 error increases by multiple testing. When focusing on DM itself, concluding the impact of an aspirin-free strategy based on bleeding events within 1-month seems to be too short. As was aforementioned, the influence of DM on a post-PCI state is not a short-term effect. Longer term data are essential to understand the impact of a softened antiplatelet strategy in patients with a high cardiovascular risk. Another important point regards the thrombotic complications. Indeed, STOPDAPT-3, as most of these trials, is not powered for thrombotic complications, particularly for those rare as stent thrombosis, and this issue becomes even more relevant in subgroup analyses which are underpowered by definition. In the overall STOPDAPT-3, the prasugrel monotherapy arm was associated with an excess of any unplanned coronary revascularization and a three-fold greater rate of subacute definite or probable stent thrombosis despite PCI was optimized by intravascular imaging in 93% of patients.^12^ In the present analysis, this finding is consistent in patients with or without DM, thus representing a concern in an era in which stent thrombosis is even more rare than in the past. Last but not least, the geographic aspect (Japanese only population) and the unique dose of prasugrel used in this study (20 mg as loading dose, then 3.75 mg daily) should be kept in mind because it affects the generalizability of such findings.

Overall, the current study explored the interaction of 1-month prasugrel monotherapy and DM, during a 1-month period after PCI. No interaction was shown in the study results, but more evidence and longer follow-up is needed to confirm the safety and efficacy of this aspirin-free strategy, particularly in patients with DM who are at increased risk of ischaemic complications over the time.

## Conflict of interest

none declared.
